# PPM1D activity promotes the replication stress caused by cyclin E1 overexpression

**DOI:** 10.1002/1878-0261.13433

**Published:** 2023-10-16

**Authors:** Andra S. Martinikova, Miroslav Stoyanov, Anna Oravetzova, Yannick P. Kok, Shibo Yu, Jana Dobrovolna, Pavel Janscak, Marcel van Vugt, Libor Macurek

**Affiliations:** ^1^ Laboratory of Cancer Cell Biology, Institute of Molecular Genetics Czech Academy of Sciences Prague Czech Republic; ^2^ Department of Medical Oncology, University Medical Center Groningen University of Groningen The Netherlands; ^3^ Department of Pathology and Medical Biology, University Medical Center Groningen University of Groningen The Netherlands; ^4^ Institute of Molecular Cancer Research University of Zurich Switzerland

**Keywords:** cancer, cell cycle, cyclin E1, PPM1D phosphatase, replication stress

## Abstract

Oncogene‐induced replication stress has been recognized as a major cause of genome instability in cancer cells. Increased expression of cyclin E1 caused by amplification of the *CCNE1* gene is a common cause of replication stress in various cancers. Protein phosphatase magnesium‐dependent 1 delta (PPM1D) is a negative regulator of p53 and has been implicated in termination of the cell cycle checkpoint. Amplification of the *PPM1D* gene or frameshift mutations in its final exon promote tumorigenesis. Here, we show that PPM1D activity further increases the replication stress caused by overexpression of cyclin E1. In particular, we demonstrate that cells expressing a truncated mutant of PPM1D progress faster from G1 to S phase and fail to complete licensing of the replication origins. In addition, we show that transcription–replication collisions and replication fork slowing caused by *CCNE1* overexpression are exaggerated in cells expressing the truncated PPM1D. Finally, replication speed and accumulation of focal DNA copy number alterations caused by induction of *CCNE1* expression was rescued by pharmacological inhibition of PPM1D. We propose that increased activity of PPM1D suppresses the checkpoint function of p53 and thus promotes genome instability in cells expressing the *CCNE1* oncogene.

## Graphical abstract

Under basal conditions, MDM2 and PPM1D suppress p53, allowing normal proliferation. *CCNE1* amplification causes premature G1/S transition, increased transcription–replication conflicts and slows down replication. This effect is partially balanced by p53. Increased activity of PPM1D suppresses p53, allowing accelerated G1/S transition and further impairing S phase progression. PPM1D inhibition allows p53 reactivation and rescues replication fork progression.

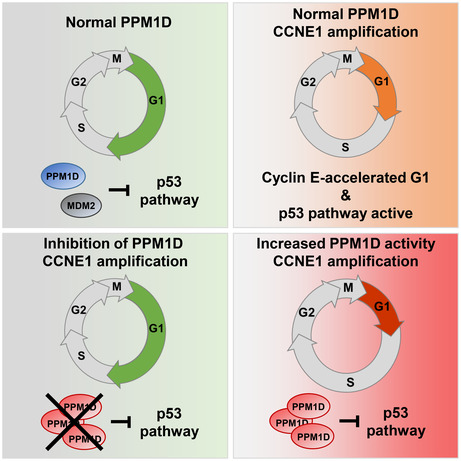

**Abbreviations**
14‐3‐3: 14‐3‐3 protein
5‐EdU: 5‐ethynyl‐2′‐deoxyuridine
A.U.: arbitrary unit
BrdU: 5′‐bromo‐2′‐deoxyuridine
CCNE1: cyclin E1
CldU: 5‐chloro‐2′‐deoxyuridine
CNA: copy number alterations
DAPI: 4′,6‐diamidino‐2‐phenylindole
DMEM: Dulbecco's modified Eagle medium
ECL: enhanced chemiluminescence
HCl: hydrochloric acid
HU: hydroxyurea
IdU: 5‐iodo‐2′‐deoxyuridine
MCM2/4/6: DNA replication licensing factor MCM2/4/6
NZ: nocodazole
PCNA: proliferating cell nuclear antigen
PFA: formaldehyde
PLA: proximity ligation assay
PPM1D: protein phosphatase magnesium‐dependent 1 delta
RPA2: replication protein A 32 kDa subunit
RPE1: retinal pigment epithelial cells
RT: room temperature
SD: standard deviation
TCGA: The Cancer Genome Atlas
TFIIH: general transcription and DNA repair factor IIH helicase subunit XPB
TP53: tumour protein p53
TRCs: transcription‐replication conflicts

## 1 Introduction

Genome instability is one of the hallmarks of cancer cells and impaired DNA replication (referred to as replication stress) caused by activation of oncogenes, has recently been identified as a common driver of genome instability [1, 2, 3, 4]. For instance, amplification of the *CCNE1* gene is frequently observed in various tumours, including high‐grade serous ovarian cancer and triple‐negative breast cancer [5, 6, 7]. Overexpression of Cyclin E1 leads to induction of DNA replication stress due to premature entry into S phase, uncontrolled replication origin firing, depletion of the nucleotide pool and collisions between replication and transcription [7, 8, 9, 10, 11, 12]. Upon stalling of replication forks, exposed single‐stranded DNA is coated by RPA complex and ATR/CHK1 kinases are activated to prevent firing of additional replication origins. Upon excessive replication stress, the cellular pool of RPA complex is exhausted, stalled forks collapse and eventually form DNA double‐stranded breaks [13]. In addition, under‐replicated DNA can interfere with chromosomal segregation during mitosis, which can lead to the formation of micronuclei and subsequent loss of genomic DNA or complex genomic rearrangements [14, 15].

Activation of the p53 pathway protects genome integrity at multiple levels. First, p53 is stabilized by active ATM kinase and governs expression of the potent CDK inhibitor p21 that slows down progression through G1 phase to allow time for DNA repair [16, 17]. Similarly, p53 has been reported to regulate the length of G1 phase to allow completion of origin licensing [18, 19]. In addition, p53 has recently been shown to promote the progression of replication forks and to facilitate resolution of stalled forks [20, 21, 22]. Multiple studies demonstrated that p53 and its transcriptional target p21 efficiently protect cells from the deleterious consequences of cyclin E1 overexpression and *TP53* gene is commonly mutated in cancers carrying amplification of the *CCNE1* locus [8, 14, 23, 24].

Protein phosphatase magnesium‐dependent 1 delta (PPM1D, also referred to as WIP1) is an established negative regulator of p53 that promotes recovery from a G2 cell cycle checkpoint [25, 26, 27]. Amplification of the genomic locus 17q23 coding for *PPM1D* or production of a highly stable and enzymatically active mutant of PPM1D have been reported in breast and ovarian cancer, therapy‐induced acute myeloid leukaemia (t‐AML) and other haematological malignancies [28, 29, 30, 31]. We and others have shown that cells expressing a truncated PPM1D mutant fail to induce a cell cycle checkpoint arrest following induction of DNA double‐strand breaks and escape programmed cell death [30, 32, 33, 34]. Here, we investigated the contribution of PPM1D activity to the cellular response to replication stress. We found that PPM1D activity suppressed the p53 pathway and promoted proliferation of RPE1 cells with inducible overexpression of the cyclin E1 oncogene. Cells co‐expressing cyclin E1 and truncated PPM1D progressed faster from G1 to S phase than cells expressing cyclin E alone, failed to complete origin licensing and showed increased number of transcription‐replication collisions. Consequently, PPM1D activity impaired progression through S phase and increased the level of replication stress. Conversely, inhibition of PPM1D restored the origin licensing, progression through the S phase and prevented genome instability. We propose that PPM1D activity promotes genome instability caused by the overproduction of cyclin E1.

## 2 Materials and methods

### 2.1 Cell lines

hTERT‐immortalized human retinal pigmented epithelial cells (RPE1, RRID:CVCL_4388, obtained from ATCC, Manassas, VA, USA), human osteosarcoma U2OS cells (RRID:CVCL_0042, obtained from Rene Medema, NKI) and all their derivatives were grown in DMEM containing 6% FBS (Gibco, Thermo Fisher Scientific, Waltham, MA, USA), Penicillin (100 U·mL^−1^) and Streptomycin (0.1 mg·mL^−1^). U2OS‐PPM1D‐KO cells with inactivated *PPM1D*; RPE1‐*TP53*‐KO cells with inactivated *TP53*; RPE1‐PPM1D‐cr1, RPE1‐PPM1D‐cr2 and RPE1‐PPM1Dtal1 cells carrying a frameshift mutation in exon 6 of *PPM1D* were described previously [32, 35]. RPE1‐pRetroX‐Tet‐On Advanced cells stably transfected with pRetroX‐Tight‐Pur‐*CCNE1* (referred to as RPE1‐cE) allowing for doxycycline‐inducible expression of cyclin E1, and RPE1‐cE‐p53^−/−^ with inactivated p53 were described previously [14]. To generate a truncating mutation in exon 6 of *PPM1D*, we transfected the inducible RPE1‐cyclin E1 cells with pSpCas9‐(BB)‐2A‐Puro plasmid (ID48139; Addgene, Watertown, MA, USA) carrying previously validated targeting sequences GAAGGCATTGCTACGAACCAGGG (CR1) or ATAGCTCGAGAGAATGTCCAAGG (CR2) and expanded the single cell clones [32]. Correct editing in the targeted region was confirmed by sequencing of the genomic DNA and the stabilization of PPM1D protein by immunoblotting. For cyclin E1 induction, the RPE1‐cE cells were treated with doxycycline (1 μg·mL^−1^) for the indicated time intervals. U2OS‐cyclin E tetOFF cells (obtained from Jiri Lukas, University of Copenhagen) were routinely cultured in the presence of doxycycline (2 μg·mL^−1^) and cyclin E expression was induced by switching to tetracycline‐free media for indicated times [3]. All cell lines were regularly checked for mycoplasma contamination and authenticated using STR profiling.

### 2.2 Antibodies and reagents

The following primary antibodies were used: PPM1D (sc376850), cyclin E1 (sc247), p21 (sc6246, clone F‐5), p53 (sc6243), 14‐3‐3 (sc133233), TFIIH (sc293), MCM4 (sc28317) and MCM6 (sc393618) from Santa Cruz Biotechnology (Dallas, TX, USA); Chk2‐pThr68 (#2197), p53‐pS15 (#9284), Chk1‐pS317 (#12302), β‐actin (#8457), Caspase‐3 Cleaved (#9664, clone 5A1E) from Cell Signaling Technology (Danvers, MA, USA); RPA2‐pS4/8 (PLA0071), γH2AX‐pS319 (clone JBW301, #05‐636) from Merck (Darmstadt, Germany); MCM2 (ab95361), R2p53/RRM2B (ab154194), proliferating cell nuclear antigen (PCNA; ab18197) and BrdU (ab6326) from Abcam (Cambridge, UK); BrdU (347580) from BD Bioscience (New Jersey, NJ, USA) and RNA polymerase II (920204, clone H5) from BioLegend (San Diego, CA, USA). HRP‐conjugated secondary antibodies for immunoblotting were from Bio‐Rad Laboratories (Hercules, CA, USA) and ECL substrate from Thermo Fisher Scientific. Alexa Fluor azide and 5′‐ethynyl‐2′‐deoxyuridine (5‐EdU) were from Jena Bioscience (Jena, Germany). GSK2830371 and nutlin‐3 were from MedChem Express (Monmouth Junction, NJ, USA) and were dissolved in DMSO.

### 2.3 Cell survival assay

Cells were seeded in 96‐well plates (100 cells/well) and after 24 h, they were treated or not with hydroxyurea (HU; 0.1 mm) for 8 days. Then, cells were incubated with resazurin (30 μg·mL^−1^; Merck) at 37 °C for 1–5 h. The emitted fluorescence at λ = 590 nm was measured using an EnVision fluorometer (Perkin Elmer, Waltham, MA, USA). The measured values were normalized to the nontreated cells of the same genotype.

### 2.4 Flow cytometry and cell cycle analysis

For determination of the cell cycle profile, cells were cultured as indicated and were incubated with 5‐EdU (10 μm) for 30 min prior harvesting by trypsinization. Cells were fixed in 4% formaldehyde and permeabilized with 0.5% Triton X‐100 for 15 min. After blocking with BSA, the click‐it reaction was performed using Alexa Fluor azide. Next, cells were stained with pMPM2‐Cy5 (Millipore, Burlington, MA, USA; #16‐220) and with 4′,6‐diamidino‐2‐phenylindole (DAPI, 5 μg·mL^−1^). Signal in single cells was measured using LSRII flow cytometer (BD Biosciences) and analysed by flowjo v10.6 program (BD Life Sciences, Ashland, OR, USA). More than 10^4^ cells were measured in each experiment and fractions of G1 (2n, EdU−), S (EdU+), G2 (4n, EdU−) and mitotic (MPM2+) cells were determined. Level of the chromatin‐bound MCM2 was determined as described [19]. Briefly, cells were pre‐extracted with Cytoskeletal buffer, fixed in 70% EtOH and stained with MCM2 antibody and subsequently with Alexa‐conjugated secondary antibody. For determining the dynamics of the S phase entry, RPE1‐cE, RPE1‐cE‐PPM1D^T1^ and RPE1‐cE‐PPM1D^T2^ cells were treated or not with doxycycline and were synchronized in mitosis by incubation with nocodazole for 12 h, collected by a mitotic shake‐off, washed two times with PBS and seeded in six‐well plates in media supplemented with EdU and with DMSO, GSK2830371 (3 μm) or Nutlin‐3 (9 μm). Cells were collected in 2‐h intervals and a fraction of EdU‐positive cells was determined by flow cytometry.

### 2.5 Immunofluorescence microscopy

Cells grown on coverslips were fixed with 4% PFA for 15 min and were permeabilized with 0.1% Triton X‐100 for 10 min at room temperature (RT). Fixed coverslips were blocked in 1% BSA for 30 min and incubated for 3 h with the primary antibodies, followed by incubation with Alexa‐conjugated secondary antibodies for 1 h. The nuclei were DAPI‐stained, and the coverslips were fixed with Vectashield (Vector Laboratories, Newark, CA, USA) on slides. For the 53BP1 and γH2AX foci evaluation, the images were acquired on Olympus ScanR high‐throughput microscope equipped with a UPLFLN 40×/1.3 OIL objective and a motorized stage. Nuclear foci were quantified using Spot Detector module. For MCM2, MCM4 and MCM6 staining, cells were incubated in pre‐extraction buffer (0.5% Triton X‐100, 25 mm Hepes pH 7.7, 50 mm NaCl, 1 mm EDTA, 3 mm MgCl_2_, 300 mm sucrose) for 5 min on ice and then fixed with PFA. Where indicated, cells were treated with EdU for 30 min prior to fixation and EdU was stained using click‐it reaction.

### 2.6 DNA fibre assay

RPE1‐cE, RPE1‐cE‐PPM1D^T1^ and RPE1‐cE‐PPM1D^T2^ were treated or not with doxycycline for 3 days, and with 1 μm PPM1Di for last 48 h or 6 μm nutlin‐3 for last 6 h before harvest. U2OS‐cyclin E tetOFF cells were treated or not with doxycycline for 3 days and 1 μm PPM1Di for last 48 h before harvest. Cells were labelled with 30 μm 5‐chloro‐2′‐deoxyuridine (CldU, C6891; Merck) for 30 min, thoroughly washed three times with warm PBS and then labelled with 250 μm 5‐iodo‐2′‐deoxyuridine (IdU, I7125; Merck) for 30 min. Cells were subsequently harvested with trypsinization, resuspended in the ice‐cold PBS and diluted to a concentration 2.5 × 10^5^ mL^−1^. To spread DNA on the slide, 2.5 μL of the labelled cells and 7.5 μL of lysis buffer (200 mm Tris–HCl (pH 7.5), 50 mm EDTA, 0.5% (w/v) SDS) were mixed directly on a slide. Slides were left to dry for 5 min and then tilted to an angle of 30° letting drop slide down and spread DNA. The spreads were air‐dried and fixed in methanol:acetic acid solution (3 : 1) for 20 min at RT. Slides were washed with PBS and then denatured in 2.5 m HCl for 1 h at RT. Residues of HCl were removed by extensive washing with PBS and slides were blocked with blocking solution (2% BSA, 0.1% Tween 20 in PBS) for 20 min. Slides were then incubated with primary antibodies rat anti‐CldU (ab6326; Abcam; IF, 1 : 500) and mouse anti‐IdU (347580; BD Biosciences; 1 : 100) diluted in the blocking solution for 2.5 h in the dark at RT. After antibody staining, slides were washed four times with PBS supplemented with 0.2% Tween‐20 (PBST) and incubated with secondary antibodies donkey anti‐rat Cy3 (712‐166‐153; Jackson ImmunoResearch, Cambridgeshire, UK; 1 : 150) and goat anti‐mouse Alexa 488 (A11001; Thermo Fisher Scientific; 1 : 300) for 2 h in the dark at RT. Slides were washed with PBST, air‐dried and mounted with a 25 μL Fluoromount‐G mounting medium (00‐4958‐02; Thermo Fisher Scientific). Images were acquired on Leica DM6000 fluorescent microscope (Wetzlar, Germany) (63×/1.40 oil immersion) and CldU and IdU tract lengths were quantified using imagej (https://imagej.net).

### 2.7 Single‐cell genome analysis

Parental RPE1, RPE1‐cE and RPE1‐cE‐PPM1D^T2^ cells were treated with mock, doxycycline or a combination of doxycycline with PPM1Di for 3 days and the G1 population was single‐cell sorted into 96‐well plates (48 cells per sample) based on a Hoechst/Propidium iodide double staining. Bravo automated liquid handling platform (Agilent Technologies, Santa Clara, CA, USA) was used to perform sample preparation and generate Illumina‐based libraries as described previously [36]. The libraries were sequenced on a NextSeq 500 sequencer (Illumina, San Diego, CA, USA) and analysed using aneufinder software as previously described [37]. Nontreated RPE1 cells were employed as a reference to determine the most frequently observed copy number state per 1 Mb and focal copy number alterations (CNAs) in individual cells were scored. Average number of the identified CNAs in all indicated cell lines was compared using the Kruskal–Wallis test.

### 2.8 *In situ* proximity ligation assay

Conflicts between transcription and replication were quantified by *in situ* proximity ligation assay (PLA) as previously described [38]. RPE1‐cE and RPE1‐cE‐PPM1D^T1^ grown on coverslips were treated or not with doxycycline for 3 days, washed with PBS and pre‐extracted for 10 min with ice‐cold CSK buffer [25 mm HEPES (pH 7.7), 50 mm NaCl, 1 mm EDTA, 3 mm MgCl_2_, 300 mm sucrose, 0.5% Triton X‐100] supplemented with a protease inhibitor cocktail (Complete, EDTA‐free; Sigma‐Aldrich, St. Louis, MI, USA) and phosphatase inhibitors cocktail (PhosSTOP™; Roche, Basel, Switzerland). Cells were subsequently washed once with CSK buffer without Triton‐X100 and fixed with 4% formaldehyde for 15 min at RT. After fixation, cells were washed twice with PBS and additionally fixed with −20 °C methanol for 20 min. Fixed coverslips were blocked in 3% BSA in PBS for 40 min and then incubated with PCNA and RNA polymerase II antibodies at 4 °C overnight. After washing with PBS, PLA was performed using Duolink flow PLA Mouse/Rabbit Kit (DUO94102‐1KT; Sigma‐Aldrich) according to the manufacturer's instructions. Coverslips were stained with DAPI and mounted using Fluoromount aqueous mounting medium (F4680; Sigma‐Aldrich). Images were acquired using Olympus IX83 microscope (Tokyo, Japan) and analysed by Olympus ScanR analysis program. PLA signal was determined in more than 500 nuclei per condition.

### 2.9 Bioinformatic analysis of cancer databases

Copy number alterations data, and mutation data of 10 967 cancer samples in the TCGA Pancancer, were obtained from cBioportal (https://www.cbioportal.org/). The CNA data were thresholded with the following cut‐off: −2 = homozygous deletion; −1 = hemizygous deletion; 0 = neutral/no change; 1 = gain; 2 = high‐level amplification. We defined gain and high‐level amplification as CNA gain states for CCNE1 and PPM1D in this study for TCGA samples. For breast cancer sample analysis, we analysed unique samples in the collective ‘Invasive Breast Carcinoma’ data set from cBiortal (*n* = 5416). Mutation status and amplification status were used as defined by cBioportal.

## 3 Results and Discussion

### 3.1 PPM1D provides a proliferation advantage upon induction of replication stress

**Fig. 1:**
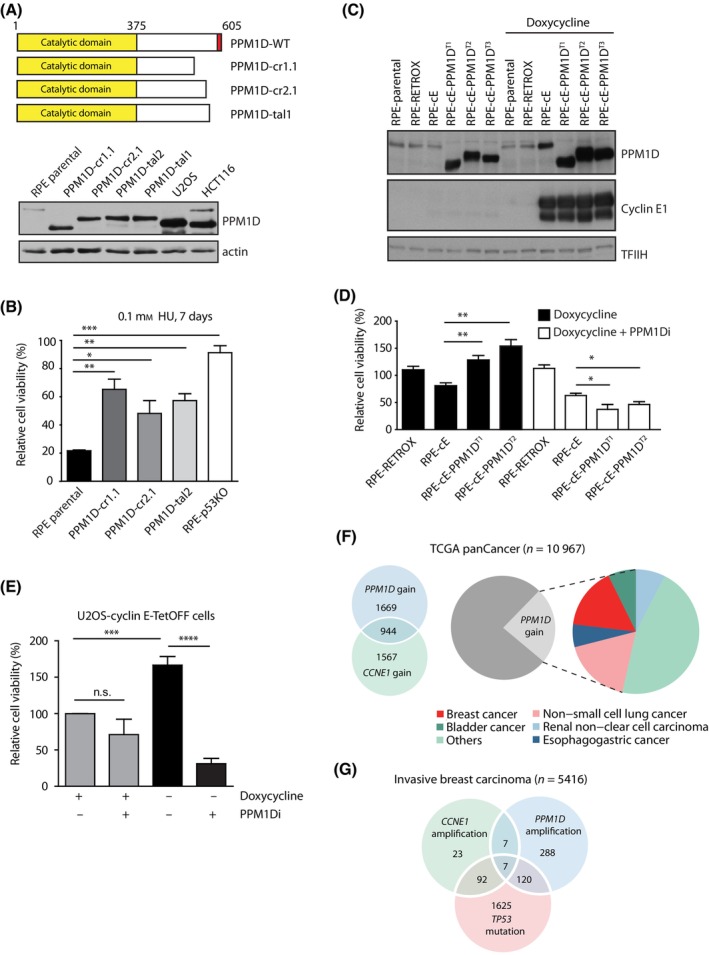
PPM1D provides proliferation advantage upon induction of replication stress. (A) Scheme of the RPE1‐PPM1D mutants used in the study. Red rectangle shows localisation of a degron responsible for degradation of PPM1D protein. Note an increased level of the truncated PPM1D in all clones detected by immunoblot of the whole cell extracts. U2OS and HCT116 cells naturally containing the truncated PPM1D alleles are used as controls (*n* = 1). (B) Parental RPE1 cells, cells carrying truncated PPM1D, and RPE1‐p53KO cells were treated with 0.1 mm HU for 7 days and relative proliferation was determined by resazurin assay. Proliferation was normalized to a condition without HU for each genotype. Statistical significance was evaluated by the two‐tailed *t*‐test, error bars indicate SDs (*n* = 3) (*P < 0.05; **P < 0.01; ***P < 0.001). (C) Parental RPE1, RPE1 cells stably transfected with pRetroX‐Tet‐On alone (RPE1‐Retrox) and with pRetroX‐Tight‐Pur‐*CCNE1* (RPE1‐cE) cells and their three independent derivatives carrying truncated PPM1D^T^ allele (RPE1‐cE‐PPM1D^T1^, RPE1‐cE‐PPM1D^T2^ and RPE1‐cE‐PPM1D^T3^) were treated or not with doxycycline for 3 days. Whole cell lysates were probed with indicated antibodies (*n* = 2). Note an increased level of the truncated PPM1D in all clones. (D) RPE1‐cE, RPE1‐cE‐PPM1D^T1^ and RPE1‐cE‐PPM1D^T2^ cells were treated or not with doxycycline or a combination of doxycycline and PPM1Di and proliferation was determined after 8 days using resazurin assay. Proliferation was normalized to a condition without doxycyline for each genotype. Statistical significance was evaluated by the two‐tailed *t*‐test, error bars indicate SDs (*n* = 4) (*P < 0.05; **P < 0.01). (E) U2OS‐cyclin E‐TetOFF cells were grown in the presence of doxycycline or expression of cyclin E was induced by removal of doxycycline. Where indicated, cells were treated with PPM1Di. Proliferation was determined after 8 days using resazurin assay. Statistical significance was evaluated by the two‐tailed *t*‐test, error bars indicate SDs (*n* = 3) (***P < 0.001; ****P ≤ 0.0001). (F) Analysis of TCGA pan‐cancer data set (*n* = 10 967 unique samples), with the pie chart showing the distribution of top five tumour types over samples with *PPM1D* gain. Venn diagram shows the intersection of samples with *PPM1D* and *CCNE1* copy number gain. (G) Analysis of cBioportal Invasive Breast Cancer samples (*n* = 5416 unique samples). Venn diagram indicates intersection of samples with *CCNE1* amplification, *PPM1D* amplification and *TP53* mutation.

High expression of PPM1D caused by amplification of the 17q23 locus in cancer cells impairs sensitivity to chemotherapy [39]. Similarly, cells carrying a C‐terminally truncated PPM1D are resistant to ionizing radiation and various DNA damage‐inducing agents, including cytarabine, etoposide and cisplatin [32, 34, 40]. Here, we aimed at testing the impact of PPM1D activity on the cellular response to replication stress, which is recognized as a major cause of genomic instability during tumour development [41, 42]. To this end, we used human nontransformed RPE1 cells and their previously characterized subclones carrying the frameshift mutations in exon 6 of the *PPM1D* gene that result in the stabilization of an enzymatically active PPM1D (Fig. 1A,B) [32]. As expected, treatment with a mild dose of HU (0.1 mm) suppressed viability of the parental RPE1 cells (Fig. 1B). In contrast, RPE1 cells carrying the truncated PPM1D showed significantly enhanced survival in the presence of HU compared with the parental RPE1 cells (Fig. 1B). Similarly, RPE1‐p53KO cells lacking the p53 were resistant to the low dose of HU and showed increased viability compared with the parental RPE1 cells (Fig. 1B) [35]. To study the impact of PPM1D activity on cell viability in context of the oncogene‐induced replication stress, we used the previously validated CRISPR/Cas9 system to introduce frameshift mutations in the last exon of *PPM1D* in RPE1‐RetroX‐TetOn‐CCNE1 cells (further referred to as RPE1‐cE cells), which inducibly express cyclin E1 upon treatment with doxycycline [14]. As expected, the C‐terminal truncation of PPM1D increased the protein levels of PPM1D in three independent clones (Fig. 1C; Fig. S1A). We observed that the prolonged induction of cyclin E1 expression slightly reduced the survival of the parental cells (Fig. 1D; Fig. S1B). In contrast, RPE1 cells carrying the truncated PPM1D showed improved survival upon induction of cyclin E1 expression (Fig. 1D; Fig. S1B). Interestingly, the observed proliferation advantage was dependent on the phosphatase activity of PPM1D as treatment of the cells with the selective PPM1D inhibitor GSK2830371, hereafter referred to as PPM1Di [35, 43], suppressed the growth of cells with the truncated PPM1D (Fig. 1D; Fig. S1C). Similarly, we observed that cyclin E expression promoted proliferation of U2OS‐cyclin E‐TetOFF cells that contain a heterozygous c.1372C>T mutation within exon 6 of the *PPM1D* gene resulting in expression of the truncated PPM1D (Fig. 1E) [30]. Conversely, inhibition of PPM1D suppressed the cyclin E‐induced proliferation confirming that PPM1D activity positively regulates proliferation in cells expressing high levels of cyclin E1 (Fig. 1E).

To investigate whether PPM1D may play a role in tumorigenesis promoted by replication stress, we analysed the 10 967 cancer samples in the TCGA Pan‐cancer cohort through cBioportal database [44, 45]. Approximately 23.8% (2613/10 967) of cancer samples showed a *PPM1D* copy number gain with breast cancer and nonsmall lung cancers most frequently showing this feature (Fig. 1F). Interestingly, we found that copy number gain of PPM1D and *CCNE1* were co‐occurring in nearly 36.1% (944/2613) of samples with a *PPM1D* copy number gain. Since breast cancers frequently showed *PPM1D* gain, we studied this cancer type in more detail. Analysis of invasive breast cancer samples (*n* = 5416 samples), revealed co‐occurrence of *PPM1D* amplification in 10.85% (14/129) of tumour samples with *CCNE1* amplification. As expected from earlier studies, *CCNE1* amplification also frequently co‐occurred with *TP53* mutations (Fig. 1G). Overall, these data indicate that expression of active PPM1D provides cells with proliferation advantage in the context of replication stress and may contribute to tumorigenesis caused by amplification of the *CCNE1* oncogene.

### 3.2 PPM1D accelerates G1/S transition and prolongs S phase upon cyclin E1 overexpression

**Fig. 2:**
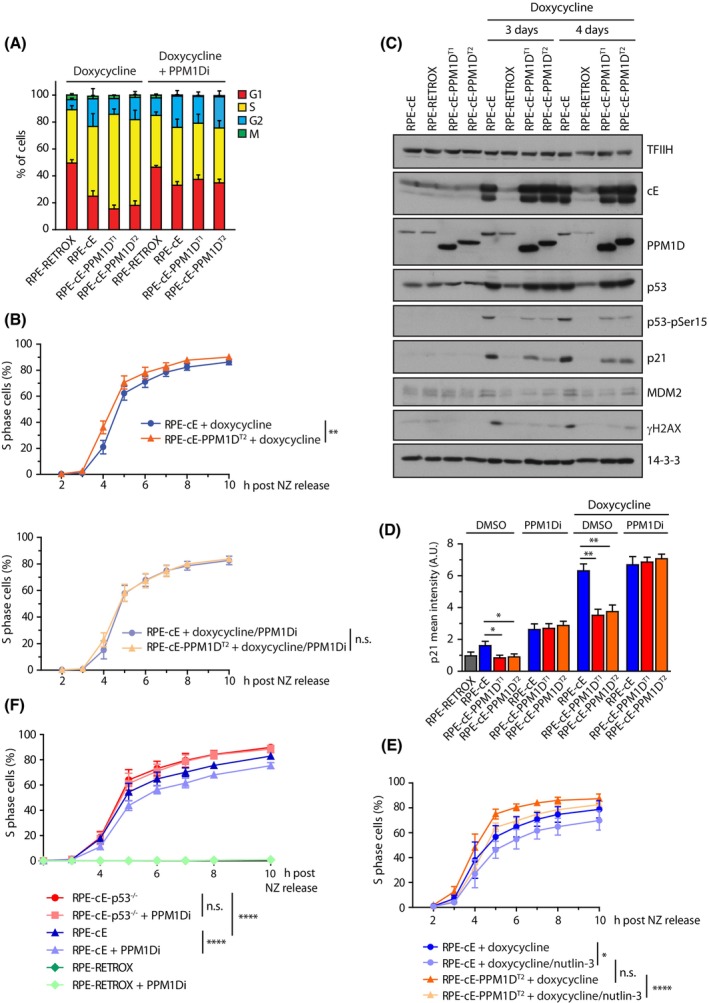
PPM1D accelerates G1/S transition and prolonges S phase upon cyclin E1 induction. (A) Parental RPE1‐Retrox, RPE1‐cE and two clones RPE1‐cE‐PPM1D^T^ cells were treated either with doxycycline or a combination of doxycycline and PPM1Di for 4 days and cells were incubated with EdU 30 min prior harvesting. After fixation, cells were probed with Mpm2 antibody, EdU was stained using click‐it reaction, DNA content with DAPI and cells were analysed by flow cytometry. Cell cycle phases were determined as 2n EdU− for G1, EdU+ for S, 4n EdU− for G2 and 4n, Mpm2+ for M. Bars indicate SD (*n* = 4). (B) RPE1‐cE and RPE1‐cE‐PPM1D^T2^ cells were synchronized in mitosis by nocodazole and were released into fresh media containing EdU and supplemented with doxycycline or combination of doxycycline and PPM1Di. Cells were collected at indicated time intervals and entry to S phase was monitored using flow cytometry as a fraction of EdU+ cells (*n* = 4). Samples were analysed in parallel but for clarity, they are presented in two separate graphs. Bars indicate SD. Statistical significance was determined by two‐way ANOVA (n.s., nonsignificant *P* > 0.05, **P < 0.01). (C) RPE1‐cE, RPE1‐cE‐PPM1D^T1^ and RPE1‐cE‐PPM1D^T2^ cells were treated with doxycycline for 3 or 4 days and whole cell lysates were analysed by immunoblotting. Representative result from two independent experiments is shown. (D) RPE1‐Retrox, RPE1‐cE, RPE1‐cE‐PPM1D^T1^ and RPE1‐cE‐PPM1D^T2^ cells were treated or not with doxycycline, PPM1Di and combination of both for 4 days. After fixation, cells were stained with p21 antibody and imaged using ScanR. Plotted is the mean nuclear intensity. More than 1000 cells were quantified per experiment, *n* = 3. Statistical significance was evaluated by the two‐tailed *t*‐test, error bars indicate SDs (*n* = 3) (**P < 0.01; *P ≤ 0.05). (E) Cells were synchronized an analysed as in B. Where indicated, nutlin‐3 (9 μm) was added to the media at time of release from the nocodazole block. Statistical significance was determined by two‐way ANOVA (*n* = 3; n.s., nonsignificant *P* > 0.05; *P ≤ 0.05, ****P ≤ 0.0001). (F) RPE1‐cE and RPE1‐cE‐p53^−/−^ cells were synchronized in mitosis by nocodazole and were released into fresh media containing EdU and supplemented with doxycycline or combination of doxycycline and PPM1Di. Entry to S phase was monitored using flow cytometry as a fraction of EdU+ cells (*n* = 4). Statistical significance was determined by two‐way ANOVA (****P ≤ 0.0001).

We have previously shown that PPM1D is required for termination of the DNA damage‐induced cell cycle arrest whereas increased PPM1D activity interferes with activation of the checkpoint [25, 30, 32]. Here, we aimed at analysing the impact of PPM1D activity on the cell cycle progression in cells experiencing replication stress. In a pilot experiment, we found that cyclin E1 expression slightly impaired replication already after 24 h of doxycycline treatment but further decrease of the replication progression was observed after extended induction of cyclin E1 expression (Fig. S2A). Immunoblotting analysis revealed a gradual increase of p21 levels between 1 and 4 days of cyclin E1 induction in RPE1‐cE cells, which is consistent with the increased replication stress leading to accumulation of DNA damage and activation of ATM/p53/p21 pathway (Fig. S2B). As expected, we observed that the doxycycline‐induced overexpression of cyclin E1 increased the fraction of the S phase cells compared to the parental RPE1‐RetroX cells, which is consistent with the slowdown of the replication forks (Fig. 2A; Fig. S2C). Moreover, we found that truncation of PPM1D further increased the fraction of S phase cells upon induction of cyclin E1 expression (Fig. 2A; Fig. S2C). Importantly, this phenotype was caused by increased activity of PPM1D as treatment with PPM1D inhibitor lowered the fraction of the S phase cells (Fig. 2A; Fig. S2C). To study the mechanism underlying the observed increase in the fraction of replicating cells, we first evaluated the dynamics of S phase entry upon induction of cyclin E1 expression. To this end, we synchronized cells in mitosis by nocodazole and released them in medium supplemented with or without doxycycline. As expected, we observed a slow and gradual increase of the fraction of S phase cells in the absence of cyclin E1 overexpression (Fig. S2D). In contrast, S phase entry was accelerated upon doxycycline‐treatment. Approximately half of the population became EdU‐positive within 4.7 h after release from the mitotic arrest, and a maximum of S phase cells was reached after 10 h (Fig. 2B, upper panel). Notably, RPE1‐cE‐PPM1D^T2^ cells entered S phase significantly faster than RPE1‐cE cells, with ~ 50% of the doxycycline‐treated RPE1‐cE‐PPM1D^T2^ being EdU‐positive at 4.4 h after release from the mitotic arrest, and with a maximum of S‐phase cells reached after 8 h (Fig. 2B). As the dynamics of S phase entry was comparable in RPE1‐cE cells and in RPE1‐cE‐PPM1D^T2^ cells treated with PPM1Di, we concluded that the rapid G1/S progression in RPE1‐cE‐PPM1D^T2^ cells was specifically promoted by PPM1D activity (Fig. 2B, lower panel). A similar effect of the truncated PPM1D on acceleration of the G1/S transition was observed in RPE1‐cE‐PPM1D^T1^ cells, further confirming that the different dynamics in the cell cycle progression was not caused by nonspecific clonal differences (Fig. S2E). We have previously described that the truncated PPM1D present in U2OS cells partially suppresses p53 pathway and impairs the G1 checkpoint [30]. As expected, U2OS cells progressed more rapidly from G1 to S phase upon induction of cyclin E1 expression (Fig. S2F) [12, 14]. Importantly, we observed that inhibition of PPM1D significantly delayed the premature entry to S phase caused by cyclin E overexpression confirming that PPM1D activity accelerates G1/S transition (Fig. S2F).

PPM1D regulates the cell cycle progression in the presence of DNA damage by suppressing the transcriptional activity of p53 [32, 46, 47]. Increased expression of the cyclin E1 has been previously shown to result in accumulation of DNA damage and activation of the p53 pathway [23, 24]. In agreement with this, we observed a massive induction of p21 expression after treatment of RPE1‐cE cells with doxycycline for 3 and 4 days (Fig. 2C,D). Whereas induction of p21 after other forms of the genotoxic stress typically leads to a G1 cell cycle checkpoint arrest, cells overexpressing cyclin E1 fail to arrest in the G1 and are apparently forced to enter S phase despite the increased level of p21, likely due to elevated CDK2 activity. As expected, cells carrying the truncated PPM1D showed lower levels of γH2AX and p53‐pS15 (Fig. 2C), which is consistent with direct dephosphorylation of H2AX and p53 by PPM1D [47, 48]. Importantly, we observed that the level of p21 was lower in cells carrying the truncated PPM1D (Fig. 2C,D; Fig. S3A), possibly explaining why these cells progress faster into S phase. In addition, RPE1‐cE‐PPM1D^T1^ and RPE1‐cE‐PPM1D^T2^ cells showed also lower levels of MDM2, another p53 target that has recently been implicated in control of replication fork progression (Fig. 2C; Fig. S3A) [22, 49].

To evaluate the contribution of p53 activity to the duration of G1 in the context of cyclin E1 induction, we compared the dynamics of the S phase entry in cells in the absence or presence of the MDM2 inhibitor nutlin‐3 [50]. We found that the accelerated entry to the S phase observed in RPE1‐cE‐PPM1D^T2^ cells was lost upon treatment with nutlin‐3, indicating that truncated PPM1D promotes the G1/S transition by suppressing the activity of p53 (Fig. 2E). Finally, we determined the dynamics of G1/S transition in RPE1‐cE and RPE1‐TP53^−/−^‐cE cells upon induction of cyclin E expression and after inhibition of PPM1D (Fig. 2F). As expected, inhibition of PPM1D delayed entry to the S phase in RPE1‐cE cells (Fig. 2F). In contrast, RPE1‐TP53^−/−^‐cE cells did not respond to inhibition of PPM1D confirming that PPM1D promotes G1/S transition by suppressing p53 pathway.

### 3.3 PPM1D activity increases the level of replication stress in cells overexpressing cyclin E1

**Fig. 3:**
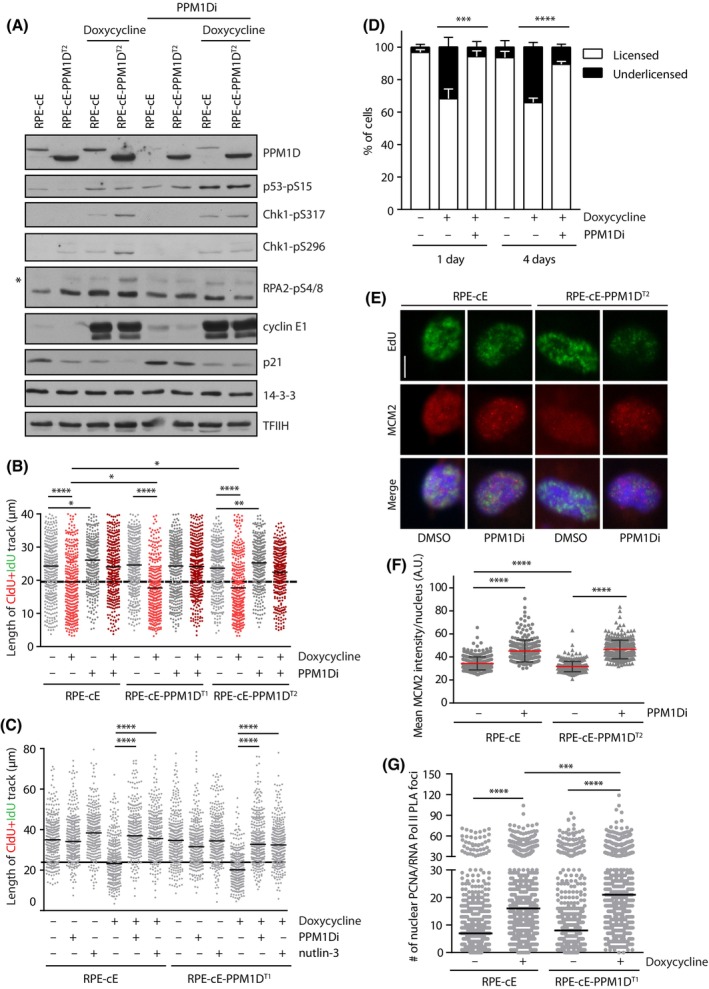
PPM1D activity increases the level of replication stress in cells overexpressing cyclin E1. (A) RPE1‐cE and RPE1‐cE‐PPM1D^T2^ cells were synchronized in mitosis by nocodazole and were released into fresh media containing EdU and supplemented with doxycycline or combination of doxycycline and PPM1Di. Cells were collected at 6‐h postrelease from nocodazole and whole cell lysates were probed with indicated antibodies. Asterisk indicates hyperphosphorylated RPA2. Staining for 14‐3‐3 and TFIIH was used as loading controls. Representative result from two independent experiments is shown. (B) Replication fork progression was determined by DNA fibre assay in RPE1‐cE, RPE1‐cE‐PPM1D^T1^ and RPE1‐cE‐PPM1D^T2^ cells treated with doxycycline, PPM1Di or combination of both as indicated. Before harvest, the cells were sequentially pulse‐labelled with CldU and IdU for 30 min each. Plotted is the length of the CldU+IdU tract, black horizontal lines indicate the mean value from three independent experiments (*n* ≥ 350). Statistical significance was determined by *t*‐test (*P ≤ 0.05; **P ≤ 0.01; ****P ≤ 0.0001). (C) RPE1‐cE and RPE1‐cE‐PPM1D^T1^ cells were treated with doxycycline, PPM1Di and nutlin‐3 as indicated and replication fork progression was determined as in B. Plotted is the length of the CldU+IdU tract, black horizontal lines indicate the mean value from three independent experiments (*n* ≥ 350). Statistical significance was determined by *t*‐test (****P ≤ 0.0001). (D) RPE1‐cE cells were treated with doxycycline and PPM1Di for 1 or 4 days and with EdU for 30 min prior extraction and fixation. The level of MCM2 in early S phase cells was determined by flow cytometry. Plotted are the fractions of cells with normal (licensed origins) or decreased level of MCM2 (underlicensed origins). Statistical significance was evaluated by the two‐tailed *t*‐test, error bars indicate SD (*n* = 4, ***P < 0.005; ****P < 0.0001). (E) RPE1‐cE and RPE1‐cE‐PPM1D^T2^ cells synchronized in mitosis by nocodazole were released into fresh medium supplemented with EdU, doxycycline and PPM1Di as indicated. After 4‐h postrelease, cells were pre‐extracted, fixed and stained for MCM2 and EdU. Cells were analysed by ScanR microscopy, representative images are shown (*n* = 3). Bar indicates 5 μm. (F) Quantification of MCM2 levels in EdU+ cells from D. Plotted is the mean nuclear intensity of MCM2 ± SD. Each dot represents a single cell (*n* = 300). Statistical significance was determined by *t*‐test. Representative out of three experiments (****P < 0.0001). (G) RPE1‐cE and RPE1‐cE‐PPM1D^T1^ cells were treated or not with doxycycline and colocalization of PCNA and the elongating form of RNA polymerase II was determined by PLA. Each dot represents a single cell (*n* = 500). Shown is representative out of three experiments. Statistical significance was determined by *t*‐test (***P < 0.001; ****P < 0.0001).

Next, we investigated the consequences of the observed accelerated S phase entry in cells expressing truncated PPM1D. The extended duration of the S phase in RPE1‐cE‐PPM1D^T2^ cells suggested that these cells may be exposed to increased replication stress compared to RPE1‐cE cells. As expected, we found that induction of cyclin E1 expression led to the activation of ATR and CHK1 kinases evaluated by the phosphorylated markers RPA2‐pS4/8, CHK1‐pS317 and CHK1‐pS296 (Fig. 3A). Interestingly, we observed even higher levels of RPA2‐pS4/8, CHK1‐pS317 and CHK1‐pS296 in RPE1‐cE‐PPM1D^T2^ compared with RPE1‐cE cells upon induction of cyclin E1 expression, which is consistent with increased replication stress (Fig. 3A). Furthermore, we found that the activity of ATR and CHK1 was comparable in RPE1‐cE and RPE1‐cE‐PPM1D^T2^ cells treated with PPM1Di, suggesting that inhibition of PPM1D protected the RPE1‐cE‐PPM1D^T2^ cells from the enhanced replication stress (Fig. 3A). Importantly, we observed that inhibition of PPM1D in U2OS cells inducibly overexpressing cyclin E decreased the activation of CHK1 (evaluated by CHK1‐pS317 and CHK1‐pS296 markers), suggesting that this phenotype is not limited to RPE1 cells (Fig. S3B).

To directly evaluate the impact of PPM1D truncation on DNA replication kinetics, we determined the progression of replication forks using DNA fiber assay. As expected, we observed a slow‐down in replication fork progression after induction of cyclin E1 expression in RPE1‐cE cells (Fig. 3B) [14]. A further decrease in replication speed was found in RPE1‐cE‐PPM1D^T1^ and RPE1‐cE‐PPM1D^T2^ cells (Fig. 3B). Importantly, in all these conditions, replication slowing was rescued by inhibition of PPM1D, confirming that PPM1D activity promotes replication stress in cyclin E1‐overexpressing cells (Fig. 3B). Similarly, we observed that induction of cyclin E1 expression suppressed the replication fork progression in U2OS cells, whereas the simultaneous inhibition of PPM1D rescued the fork speed (Fig. S3C). To determine the mechanism by which PPM1D activity promotes replication stress in cells expressing high levels of cyclin E1, we repeated the DNA fiber assay with cells treated with nutlin‐3 (Fig. 3C). Interestingly, we found that nutlin‐3 rescued the replication speed in RPE1‐cE and RPE1‐cE‐PPM1D^T1^ cells indicating that PPM1D activity promotes replication stress by suppressing p53 response.

Cells overexpressing cyclin E1 have been previously shown to enter S phase before completing the licensing of chromosomal replication origins [19, 51]. Because we observed an extreme shortening of the G1 phase in cells carrying the truncated PPM1D and overexpressing cyclin E1, we hypothesized that these cells may experience severe defects in the origin licensing. To evaluate origin licensing, we labelled asynchronically growing cells by EdU and determined the level of chromatin‐bound MCM2 in permeabilized cells by flow cytometry [19]. We observed that upon induction of cyclin E1, a fraction of cells entered to S phase with a low amount of MCM2 loaded on chromatin (Fig. 3D; Fig. S4A). Importantly, the fraction of cells with underlicensed origins was rescued when the duration of the G1 was extended by inhibition of PPM1D (Fig. 3D). Moreover, quantitative microscopy revealed a decreased nuclear MCM2 intensity after cyclin E1 induction in Edu‐positive RPE1‐cE‐PPM1D^T2^ cells compared with RPE1‐cE cells (Fig. 3E,F). In line with our observations with flow cytometry, level of the chromatin‐bound MCM2 in RPE1‐cE‐PPM1D^T2^ cells was rescued by inhibition of PPM1D, confirming that the defect in MCM2 loading was indeed caused by PPM1D activity (Fig. 3E,F). Similarly, we found that levels of the chromatin‐bound MCM4 and MCM6 were reduced in RPE1‐cE‐PPM1D^T2^ compared with RPE1‐cE cells upon induction by doxycycline and the loading was rescued by inhibition of PPM1D (Fig. S4B). We conclude that PPM1D activity promotes premature entry to S phase, prior to completion of origin licensing in G1. Impaired licensing has been previously linked with accumulation of DNA damage in heterochromatin regions [51]. In addition, impaired licensing increases the distances between active origins and accelerates progression of the replication forks [52, 53]. Therefore, other defects that cause replication fork slowing are likely present in cells with increased activity of PPM1D. Shortening of the G1 has been shown to impair inactivation of the intragenic origins resulting in transcription‐replication conflics (TRCs) during S phase [12]. To evaluate TRCs, we microscopically imaged the interaction between PCNA and active RNA polymerase II using PLA and as expected, we observed a strong increase in the nuclear signal upon induction of cyclin E1 (Fig. 3G) [9]. Interestingly, we noted a significantly increased number of the PLA foci in RPE1‐cE‐PPM1D^T1^ cells induced by doxycycline, suggesting that PPM1D activity promotes TRCs (Fig. 3G).

### 3.4 The activity of PPM1D contributes to genomic instability in cells overexpressing cyclin E1

**Fig. 4:**
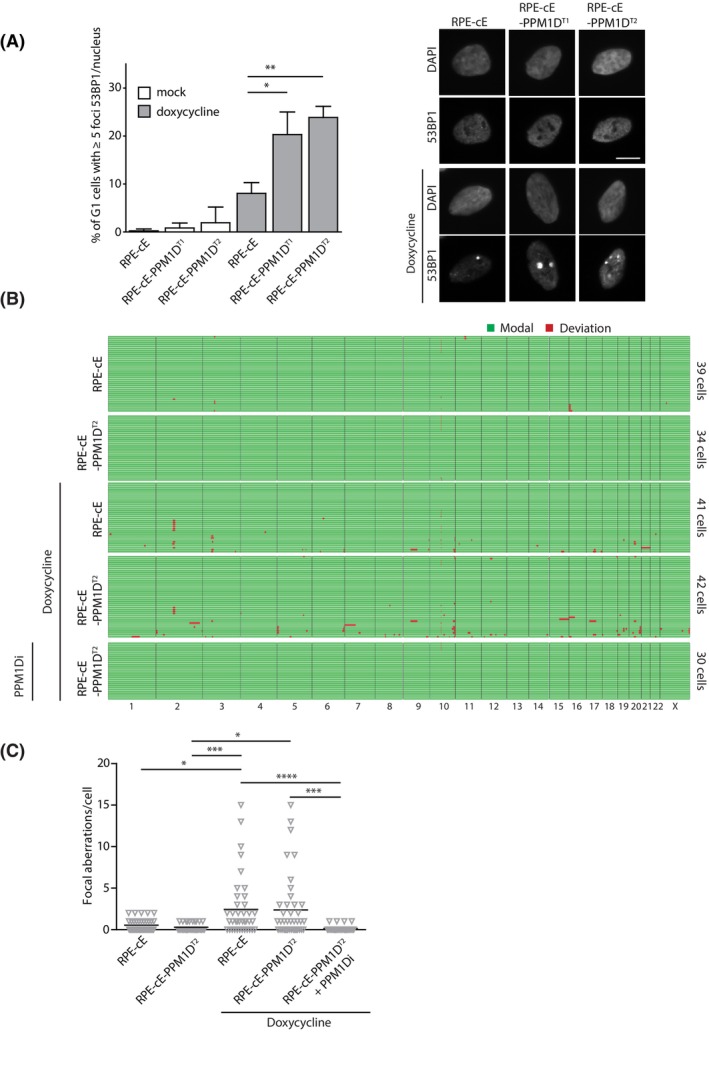
Activity of PPM1D contributes to genomic instability in cells overexpressing cyclin E1. (A) RPE1‐cE, RPE1‐cE‐PPM1D^T1^ and RPE1‐cE‐PPM1D^T2^ cells were fixed after treatment with mock or doxycycline for 3 days and with EdU for last 30 min, probed with 53BP1 antibodies and imaged using Olympus ScanR. Plotted is a population of cells with more than five 53BP1 foci in G1 cells (2n, EdU−). More than 500 cells were analysed per condition (*n* = 3). Statistical significance was evaluated by the two‐tailed *t*‐test (*P < 0.05, **P < 0.01). Representative images are shown on the right, bar indicates 1 μm. (B) RPE1‐cE and RPE1‐cE‐PPM1D^T2^ cells were treated with mock or doxycycline or a combination of doxycycline with PPM1Di for 3 days. Single cells were sequenced and the reads were analysed by aneufinder software. Every line represents data from a single cell (*n* > 30). Green indicates no difference of the focal copy number state compared to that observed in nontreated RPE1 cells, deviations are shown in red. (C) Quantification of B. Plotted is the number of focal copy number abberations per cell, bar indicates average. Statistical significance was evaluated by the Kruskal–Wallis test (*P < 0.05; ***P < 0.005; ****P < 0.0001).

Finally, we asked whether PPM1D activity contributes to genome instability in cells expressing high levels of cyclin E1. Using quantitative microscopy, we determined the number of nuclear 53BP1 bodies that assemble around DNA lesions in nascent G1 cells as a consequence of replication stress [54]. We found that the number of nuclear 53BP1 foci was elevated in RPE1‐cE cells 3 days after induction of cyclin E1 expression, suggesting that DNA damage in these cells accumulates over time (Fig. 4A). Interestingly, significantly higher fraction of RPE1‐cE‐PPM1D^T1^ and RPE1‐cE‐PPM1D^T2^ cells showed increased number of 53BP1 foci suggesting that cells with increased PPM1D activity experience higher level of DNA damage compared to RPE1‐cE cells (Fig. 4A). To directly evaluate the level of genomic instability in cell overexpressing cyclin E1, we performed single‐cell sequencing. Consistent with previous observations, we found that cyclin E1 expression elevated the load of focal DNA copy number aberrations (Fig. 4B,C) [14]. Surprisingly, we did not observe increased number of genomic abberations after 3 days of cyclin E1 overexpression in RPE1‐cE‐PPM1D^T2^ cells (Fig. 4B,C). We cannot exlude the possibility that this period of cyclin E1 overexpression was too short to reveal a positive contribution of PPM1D activity to the development of genetic instability. Nevertheless, we observed that pharmacological inhibition of PPM1D prevented development of cyclin E1‐induced genetic aberrations in RPE1‐cE‐PPM1D^T2^ cells, indicating that even a small delay of the G1/S transition may allow cells to complete replication prior to mitotic entry to safeguard genome integrity (Fig. 4B,C).

## 4 Conclusions

In summary, we report here a new mechanism that allows cells to functionally suppress the p53 pathway upon overexpression of cyclin E1, potentially promoting its pathogenic potential (Fig. S5) [8, 14, 23, 24]. In particular, we found that the activity of PPM1D phosphatase increased the replication stress in RPE1 and U2OS cells overexpressing cyclin E1. Furthermore, we observed that increased PPM1D activity caused by a truncating mutation in the last exon of *PPM1D* accelerated the progression through G1 and promoted premature entry into S phase. Based on these observations, we propose that PPM1D activity contributes to the level of replication stress by affecting the dynamics of the cell cycle progression. We provide evidence that increased activities of CDK2/cyclin E1 and PPM1D jointly trigger DNA replication prior to completing licensing of replication origins [19, 55]. In addition, we show that more transcription–replication collisions occur in cells with increased PPM1D activity likely reflecting the impaired inactivation of intragenic origins caused by extreme shortening of the G1 phase [12]. Since p53 and its transcriptional target MDM2 have been proposed to affect processivity of replication forks, it is also plausible that PPM1D may interfere with the function of p53 directly during the S phase [20, 21]. Importantly, these molecular mechanisms are not mutually exclusive and they may co‐exist in cyclin E1 overexpressing cells to allow efficient supression of the p53 function by PPM1D activity. Moreover, we report that pharmacological inhibition of PPM1D restores the normal speed of replication fork progression and prevents development of cyclin E1‐induced genetic aberrations, strongly supporting the ability of PPM1D to promote the pathogenic potential of cyclin E1 oncogene. Finally, p53 pathway has recently been implicated in promoting mitotic bypass in cyclin E1 expressing cells leading to the whole‐genome duplication [56]. At the same time, p53 activty needs to be supressed to allow proliferation of the tertaploid cells [56, 57]. As PPM1D acts as a negative regulator of p53, it will be interesting to address its potential role in cell fate decisions in cells overexpressing cyclin E1.

## Conflict of interest

The authors declare no conflict of interest.

## Author contributions

ASM generated stable cell lines and performed initial experiments, MS analysed the cell cycle progression and performed microscopic analysis, AO performed DNA fibre assays, YPK analysed the sequencing data, SY performed the bioinformatic analysis, PJ, JD, MV and LM supervised students and interpreted data, LM conceived the study and wrote the manuscript. All authors participated on data analysis and editing of the manuscript.

### Peer review

The peer review history for this article is available at https://www.webofscience.com/api/gateway/wos/peer‐review/10.1002/1878‐0261.13433.

## Supporting information


**Fig. S1.** Generation of RPE‐cE‐PPM1D^T^ cells carrying the truncated PPM1D.
**Fig. S2.** PPM1D activity affects S phase entry and duration.
**Fig. S3.** PPM1D activity supresses p53 pathway activation induced by CCNE1.
**Fig. S4.** PPM1D activity impairs licensing in CCNE1 overexpressing cells.
**Fig. S5.** Model for PPM1D function in replication stress.

## Data Availability

The source data underlying this article are available in the Suppporting Information or will be provided by the corresponding author upon request. Sequencing data were deposited in European Nucleotide Archive under accession number PRJEB55698.
