# Supplementary material for: PPM1D activity promotes the replication stress caused by cyclin E1 overexpression
Source: Mol Oncol. 2023 Oct 16;18(1):6–20. doi: 10.1002/1878-0261.13433 (PMC10766204; doi:10.1002/1878-0261.13433)

Suppl. Figure 1. Generation of RPE-cE-PPM1D^{T} cells carrying the truncated PPM1D

A) Schematic representation of the targeting of the exon 6 of *PPM1D* in RPE1-cE clones generated by CRISPR/Cas9. Note that RPE1-cE-PPM1D^{T1} clone is a compound heterozygote containing two frameshift mutations (one deletion of 7 nucleotides and one insertion of 242 nucleotides, stop codon is shown in bold). RPE1-cE-PPM1D^{T2} and RPE1-cE-PPM1D^{T3} clones are homozygotes carrying insertion of a single nucleotide generating a shift in the reading frame.

B) RPE1-Retrox, RPE1-cE, RPE1-cE-PPM1D^{T1} and RPE1-cE-PPM1D^{T2} cells were grown in doxycycline-free media and were treated with DMSO or PPM1D inhibitor for 7 days. Cell viability was determined by resazurin assay and was normalized to non-treated condition. Statistical significance was evaluated by the two-tailed t test, error bars indicate SDs (n=3, **p≤0.01).

C) RPE1-Retrox, RPE1-cE, RPE1-cE-PPM1D^{T1} and RPE1-cE-PPM1D^{T2} cells were induced or not with doxycycline and treated or not with PPM1D inhibitor for 7 days. Whole cell lysates were analyzed by immunoblotting. Cleaved caspase is a marker of apoptosis; TFIIH and 14-3-3 were used as loading controls.

Suppl. Fig. 1

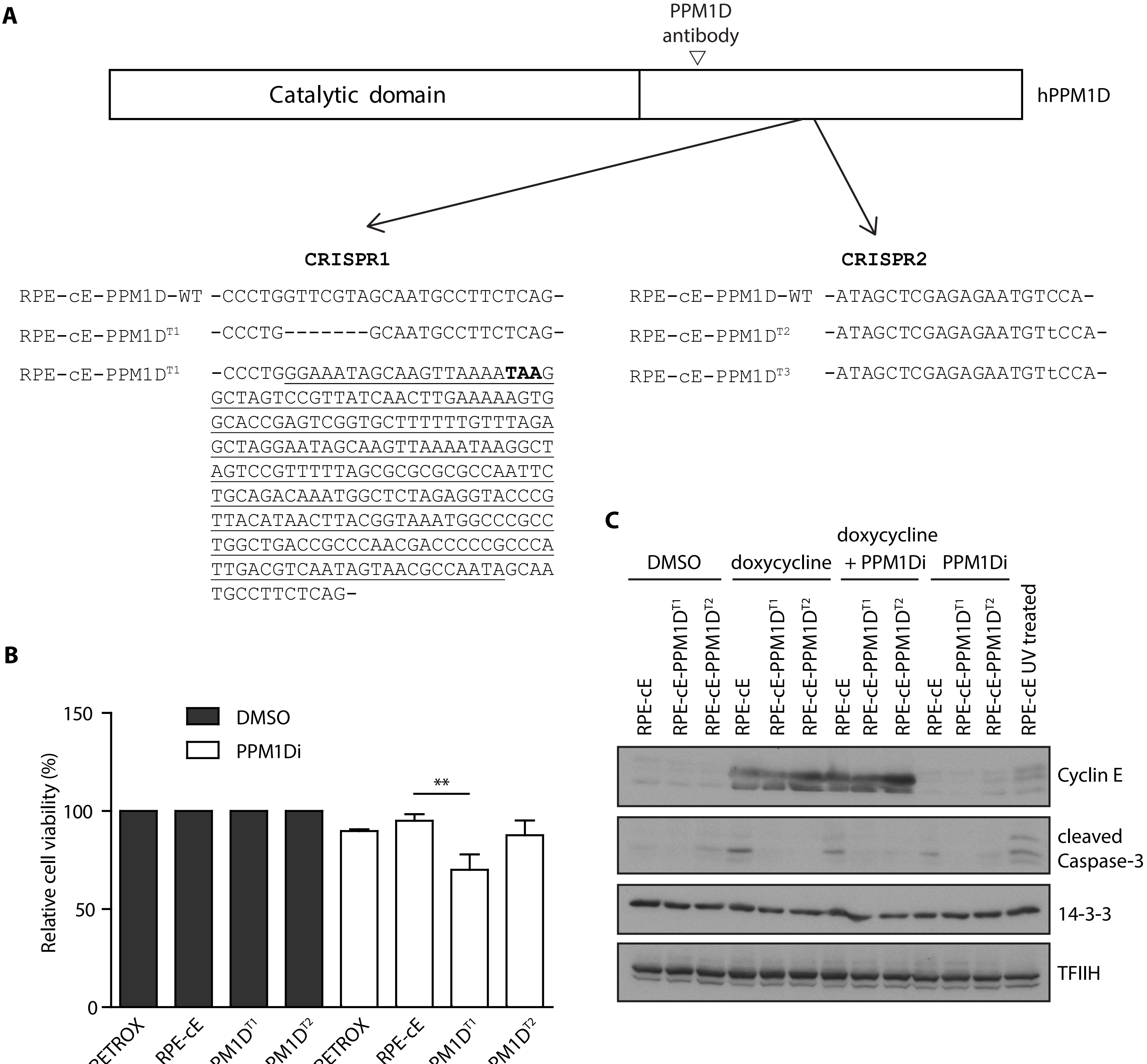
A
PPM1D antibody
Catalytic domain
hPPM1D
CRISPR1
RPE-cE-PPM1D-WT -CCCTGGTTCGTAGCAATGCCTTCTCAG-
RPE-cE-PPM1D^{T1} -CCCTG-------GCAATGCCTTCTCAG-
RPE-cE-PPM1D^{T1} -CCCTGGGAAATAGCAAGTTAAAATAAG
GCTAGTCCGTTATCAACTTGAAAAAGTG
GCACCGAGTCGGTGCTTTTTTGTTTAGA
GCTAGGAATAGCAAGTTAAAATAAGGCT
AGTCCGTTTTTAGCGCGCGCGCCAATTC
TGCAGACAAATGGCTCTAGAGGTACCCG
TTACATAACTTACGGTAAATGGCCCGCC
TGGCTGACCGCCCAACGACCCCCGCCCA
TTGACGTCAATAGTAACGCCAATAGCAA
TGCCTTCTCAG-
CRISPR2
RPE-cE-PPM1D-WT -ATAGCTCGAGAGAATGTCCA-
RPE-cE-PPM1D^{T2} -ATAGCTCGAGAGAATGTtCCA-
RPE-cE-PPM1D^{T3} -ATAGCTCGAGAGAATGTtCCA-
B
DMSO
PPM1Di
Relative cell viability (%)
150
100
50
0
**
RPE-RETROX
RPE-cE
RPE-cE-PPM1D^{T1}
RPE-cE-PPM1D^{T2}
RPE-RETROX
RPE-cE
RPE-cE-PPM1D^{T1}
RPE-cE-PPM1D^{T2}
C
DMSO
doxycycline
doxycycline + PPM1Di
PPM1Di
RPE-cE
RPE-cE-PPM1D^{T1}
RPE-cE-PPM1D^{T2}
RPE-cE UV treated
Cyclin E
cleaved Caspase-3
14-3-3
TFIIH

Suppl. Figure 2. PPM1D activity affects S phase entry and duration

A) Replication fork progression was determined by DNA fiber assay. RPE1-cE cells were treated or not with doxycycline for 1-3 days and sequentially pulse-labeled with CldU and IdU for 30 min each. Plotted is the length of the CldU+IdU tract (n≥125). Statistical significance was determined by t-test (*p≤0.05; ****p≤0.0001).

B) RPE1-cE and RPE1-cE-PPM1D^{T2} cells were treated or not with doxycycline for indicated times. Whole cell lysates were analyzed by immunoblotting.

C) RPE1-cE and RPE1-cE-PPM1D^{T2} cells were treated or not with doxycycline for 4 days. Cells were pulsed with EdU for 30 min, then cultivated for additional 8 h and finally pulsed with BrdU for 30 min prior harvesting. Fraction of cells that were replicating DNA throughout the interval was determined as EdU+/BrdU+ double positive cells using flow cytometry. Statistical significance was determined by t-test (n=3, , **p≤0.01; ***p < 0.005).

D) RPE1-cE and RPE1-cE-PPM1D^{T2} cells were synchronized in mitosis by nocodazole (NZ) and were released into fresh doxycycline-free media containing EdU. Cells were collected at indicated time intervals and entry to S phase was monitored using flow cytometry as a fraction of EdU+ cells. Representative plot from two experiments is shown. Statistical significance was determined by two-way ANOVA (****p < 0.0001).

E) RPE1-cE and RPE1-cE-PPM1D^{T1} cells were synchronized in mitosis by nocodazole and were released into fresh media containing EdU and supplemented with doxycycline or combination of doxycycline and PPM1Di. Cells were collected at indicated time intervals and entry to S phase was monitored using flow cytometry as a fraction of EdU+ cells (n=3). Statistical significance was determined by two-way ANOVA (n.s, non-significant; **p≤0.01).

F) U2OS-cyclin E-tetOFF cells were grown in the presence of doxycycline or cyclin E expression was induced by switching to tetracycline free media. Cells were synchronized in mitosis by nocodazole, collected by shake-off, washed and released to media supplemented with EdU. Cells were collected at indicated times and entry to S phase was monitored using flow cytometry as a fraction of EdU+ cells (n=2). Statistical significance was determined by two-way ANOVA (n.s, non-significant; **p≤0.01).

Supplementary Fig. 2

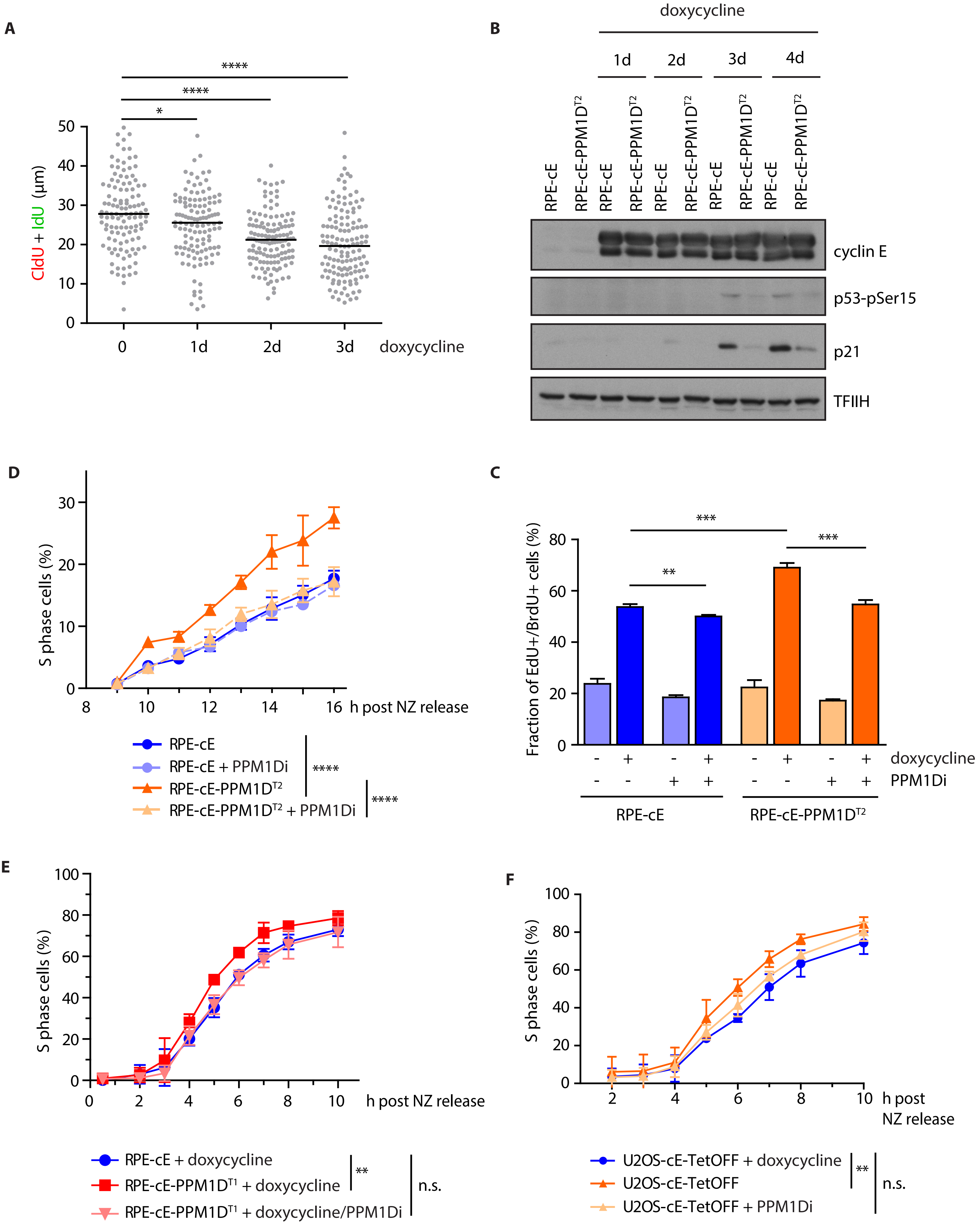

Suppl. Figure 3. PPM1D activity supresses p53 pathwaz activation induced by CCNE1

A) Quantification of the signal from Fig. 2C. Whole cell lysates of RPE1-Retrox, RPE1-cE, RPE1-cE-PPM1D^{T1} and RPE1-cE-PPM1D^{T2} cells induced with doxycycline for 3 days were analyzed by immunoblotting. Signal intensity of p21, MDM2, γH2AX and p53-pS15 from all biological replicates was measured in Image J and was normalized to the appropriate loading control. Plotted is the fold change to the RPE-Retrox cells. Statistical significance was determined by t-test (*$p \leq 0.05$; **$p \leq 0.01$; ***$p < 0.005$).

B) U2OS-cyclin E-tetOFF cells were grown in the presence of doxycycline or cyclin E expression was induced by switching to tetracycline free media. Cells were synchronized in mitosis by nocodazole, collected and released from mitosis for 6 h. Where indicated cells PPM1D inhibitor was added to the media. Whole cell lysates were collected at 6 h after nocodazole release and were analyzed by immunoblotting with indicated antibodies. Empty and full arrowhead indicate the wild type and truncated PPM1D. Staining for 14-3-3 and TFIIH served as loading control. Asterisk indicates a non-specific band.

C) U2OS-cyclin E-tetOFF cells were grown in the presence of doxycycline or cyclin E expression was induced by switching to tetracycline free media for 72 h. Where indicated, PPM1D inhibitor was added to the media for the last 48 h prior harvesting. Cells were sequentially pulse-labeled with CldU and IdU for 30 min each and harvested. Plotted is the length of the CldU+IdU tract, black horizontal lines indicate the mean. Each dot represents a single cell ($n \geq 100$) from three independent experiments ($n \geq 350$). Statistical significance was determined by t-test (****$p \leq 0.0001$).

Supplementary Fig. 3

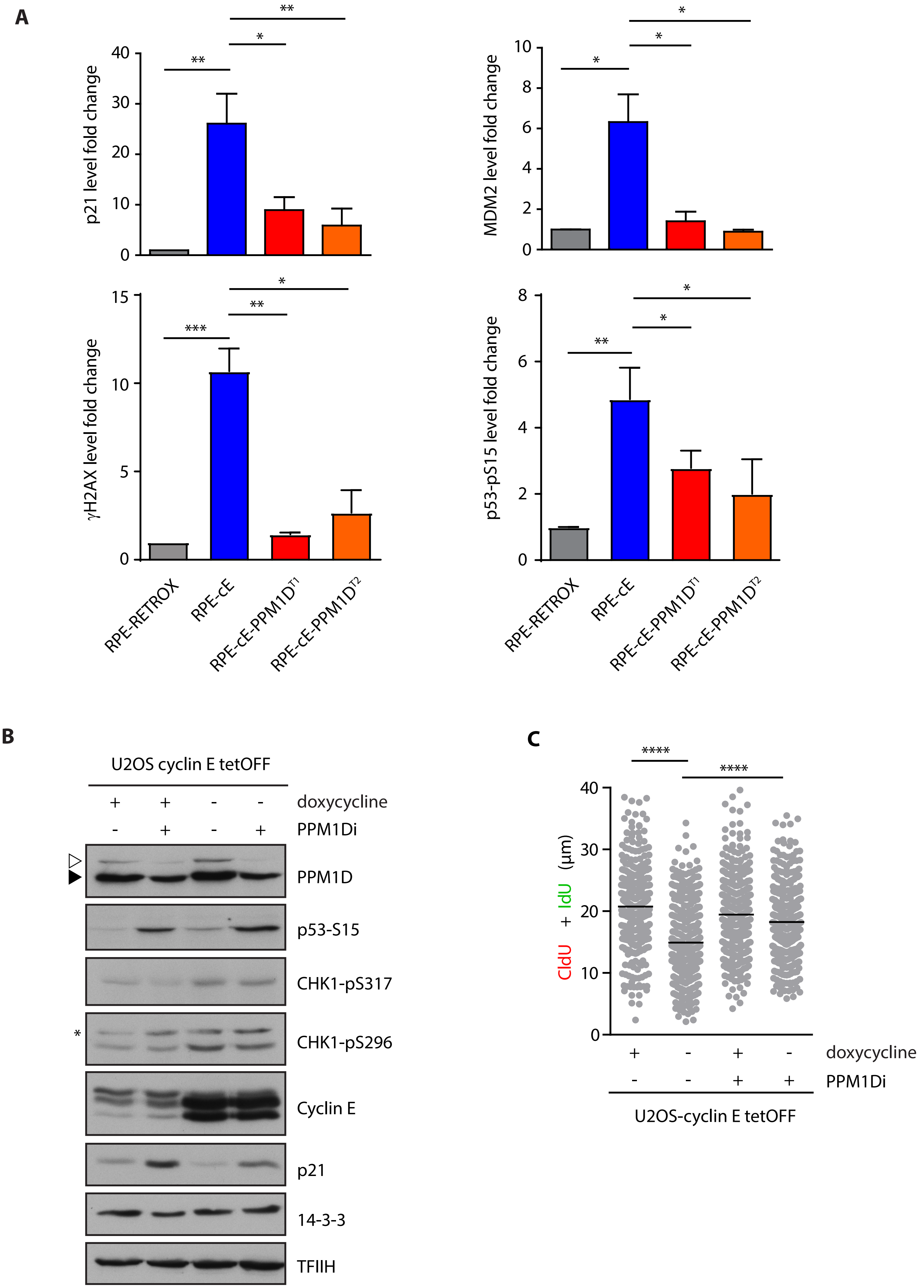

Suppl. Figure 4. PPM1D activity impairs licensing in CCNE1 overexpressing cells

A) An example of gating of the flow cytometry experiment shown in Fig. 3D. RPE1-cE cells were treated with doxycycline and PPM1Di as indicated and 30 min prior extraction with EdU. Upon fixation, cells were stained with MDM2 antibody and EdU was labeled by Click-IT reaction. Percentage of early S phase cells that contained normal (orange) or reduced (green) level of MDM2 was determined by flow cytometry. Representative images from 4 independent repeats are shown.

B) RPE1-cE and RPE1-cE-PPM1D^{T2} cells synchronized in mitosis by nocodazole were released into fresh medium supplemented with EdU, doxycycline and PPM1Di as indicated. After 4 h post-release, cells were pre-extracted, fixed and stained for MCM4 or MCM6 and EdU. Cells were analyzed by ScanR microscopy. Plotted is the mean nuclear intensity of MCM4 or MCM6 ±SD. Representative out of two experiments. Each dot represents a single cell (n=300). Statistical significance was determined by t-test (***p < 0.005; ****p < 0.0001).

A

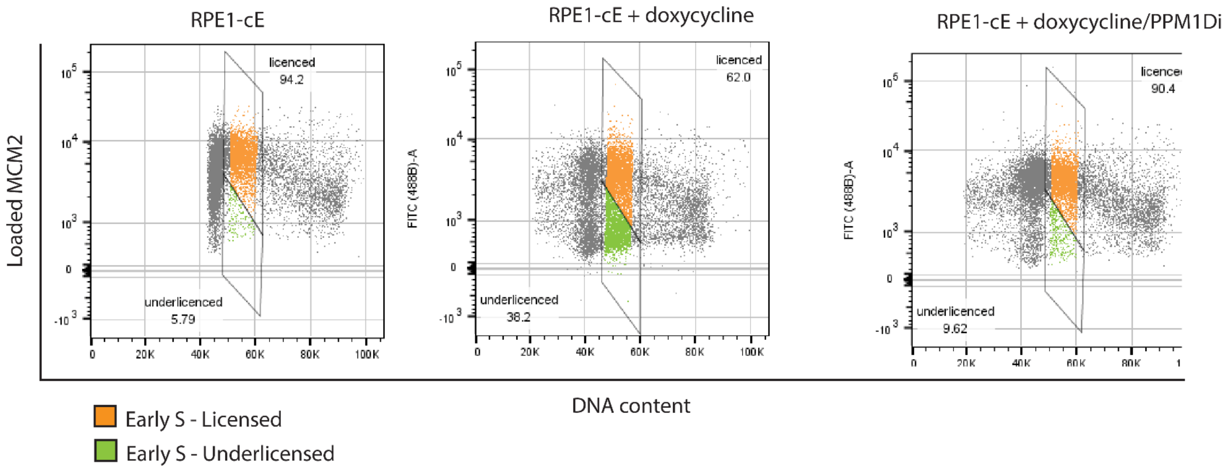

B

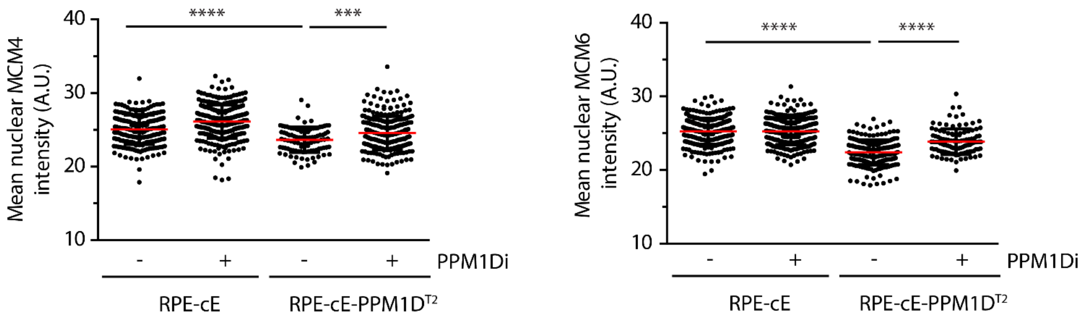

Suppl. Figure 5. Model for PPM1D function in replication stress

In the absence of DNA damage, MDM2 and PPM1D supress the stability and activity of p53 allowing normal proliferation (green panel). Increased expression of cyclin E1 causes premature G1/S transition resulting in increased transcription-replication conflicts and slowing the replication. The effect of increased cyclin E1/CDK2 activity is partially ballanced by activation of p53 that prevents extreme shortening of G1 (orange panel). Increased activity of PPM1D supresses p53 pathway allowing accelerated G1/S transition induced by high level of cyclin E1 and further impairing S phase progression (red panel). Inhibition of PPM1D leads to activation of p53, extension of G1 phase and rescuing the replication fork progression.

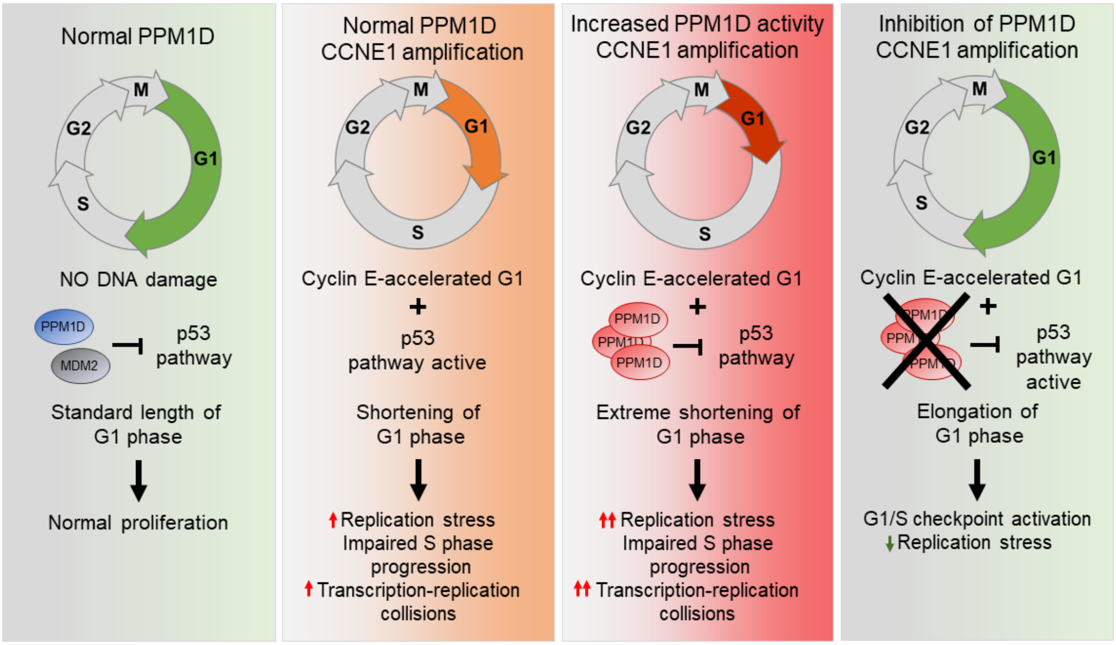

Supplement: Supplementary file 1 — Fig. S1. Generation of RPE‐cE‐PPM1DT cells carrying the truncated PPM1D. Fig. S2. PPM1D activity affects S phase entry and duration. Fig. S3. PPM1D activity supresses p53 pathway activation induced by CCNE1. Fig. S4. PPM1D activity impairs licensing in CCNE1 overexpressing cells. Fig. S5. Model for PPM1D function in replication stress. [file MOL2-18-6-s001.pdf]
